# Prioritization of COVID-19 risk factors in July 2020 and February 2021 in the UK

**DOI:** 10.1038/s43856-023-00271-3

**Published:** 2023-03-30

**Authors:** Sivateja Tangirala, Braden T. Tierney, Chirag J. Patel

**Affiliations:** 1 Department of Biomedical Informatics, Harvard Medical School, Boston, MA USA; 2 Department of Statistics and Data Science, Cornell University, Ithaca, NY USA

**Keywords:** Viral infection, Computational biology and bioinformatics

## Abstract

**Background:**

Risk for COVID-19 positivity and hospitalization due to diverse environmental and sociodemographic factors may change as the pandemic progresses.

**Methods:**

We investigated the association of 360 exposures sampled before COVID-19 outcomes for participants in the UK Biobank, including 9268 and 38,837 non-overlapping participants, sampled at July 17, 2020 and February 2, 2021, respectively. The 360 exposures included clinical biomarkers (e.g., BMI), health indicators (e.g., doctor-diagnosed diabetes), and environmental/behavioral variables (e.g., air pollution) measured 10–14 years before the COVID-19 time periods.

**Results:**

Here we show, for example, “participant having son and/or daughter in household” was associated with an increase in incidence from 20% to 32% (risk difference of 12%) between timepoints. Furthermore, we find age to be increasingly associated with COVID-19 positivity over time from Risk Ratio [RR] (per 10-year age increase) of 0.81 to 0.6 (hospitalization RR from 1.18 to 2.63, respectively).

**Conclusions:**

Our data-driven approach demonstrates that time of pandemic plays a role in identifying risk factors associated with positivity and hospitalization.

## Plain language summary

Social, demographic, and environmental factors have been shown to impact whether a person becomes infected following SARS-CoV-2 exposure. However, it is unclear whether the impact of different factors has changed as the pandemic has progressed. Here we analyze 360 factors and whether they are associated with the proportion of people being found to be infected with SARS-CoV-2 across two periods of time in the UK. Overall, we found that different risk factors were associated with testing positive for SARS-CoV-2 infection early in the pandemic compared to later in the pandemic. These results highlight that public health priorities should be adjusted as a consequence of changing risk and susceptibility to infection as the pandemic progresses.

## Abstract (web summary)

Tangirala et al. evaluate changes in the associations between various exposures and COVID-19 positivity and hospitalization across two non-consecutive waves of the pandemic in participants of the UK Biobank. They find that the strength of the association between certain risk factors, such as age and household-related factors, change over time.

## Introduction

Observational studies of COVID-19 have implicated age, sex, and sociodemographic, clinical, and environmental inequalities^1,2^. Most of this literature, however, does not consider or contextualize association sizes among the vast array of potential risk factors or take advantage of the large number of variables available in current-day biobank-scale studies, potentially missing the fuller picture of COVID-19 susceptibility. Secondly, association sizes and their strength may change due to time of sampling of COVID-19. Time of sampling may be an important parameter as, for example, certain factors may confer greater or lesser risk for COVID-19 related outcomes (e.g., COVID-19 positive test, COVID-19 hospitalization) as time progresses with newer COVID-19 variants. In fact, some studies have analyzed certain subsets of risk factors over the course of the pandemic and found associations that change over time. Roso-Llorach et al. investigated age, sex, smoking status, socioeconomic status and comorbidity burden (using the Charlson comorbidity index^3^) in association with COVID-19 hospitalization and mortality-related outcomes (e.g., 30-day mortality [dying within the 30 days following admission due to COVID-19], transfer to intensive care unit) in Catalonia, Spain, during February 2020-21. Roso-Llorach et al. assessed the differences in mortality and clinical outcomes across four successive waves and found a notable increase in the proportion of socioeconomically-deprived patients being hospitalized due to COVID-19 after the first wave^3^.

Here, to identify robust candidate observational risk factors, we perform a data-driven search for 360 correlates of COVID-19 positivity and complication across multiple time points in the pandemic (July 17, 2020 and February 2, 2021) inspired by “environment-wide association studies” (EWASs) in participants of the UK Biobank to identify how risk factors change during two different key time periods in the pandemic. Use of the EWASs can help to identify variables in large databases for prioritization of potential correlates between modifiable and non-modifiable behavior, environmental, and phenotypic factors associated with an outcome^4,5^, that may or not be investigated in a study that investigates a handful of variables at a time^6–10^. Second, we probe the variability of associations due to study design and model choice by performing the “vibration of effects” analysis^11^ for 13 of the top exposures identified from our analysis and compare this variation due to model selection with time of data collection. Overall, we found that different risk factors were associated with testing positive for SARS-CoV-2 infection early in the pandemic (e.g., frequency of shift work) compared to later (e.g., household-related factors such as presence of son and/or daughter in household) in the pandemic and age playing a more prominent role over time.

## Methods

### Study population

The UK Biobank cohort is a prospective cohort including over 500,000 participants of ages 40–69 during recruitment from 2006–2010^12^. Differences between the UK Biobank cohort individuals and the general UK population were studied by Fry et al. in order to better understand sampling “uncertainty”^13^. Their study suggested that nonparticipants are more likely to be male, younger, and live in more socioeconomically deprived areas than UK Biobank participants^13^. Information regarding how the UK Biobank data is maintained and validated can be found at https://biobank.ndph.ox.ac.uk/~bbdatan/Data_cleaning_overall_doc_showcase_v1.pdf.

We analyzed two non-overlapping subsets of the UK Biobank [UKB] cohort (total *n* = 502,628 participants) for which we had data pertaining to COVID-19 testing for tests administered until July 17, 2020 and tests administered between July 18, 2020 and February 2, 2021. COVID-19 testing in the UK was carried out in two major phases (Pillar I and Pillar II) during the time period we considered. The first phase (Pillar I) prioritized individuals with health complications and healthcare workers. We excluded participants whose ethnicity was not known, yielding samples of 9268 and 38,837 participants, respectively. The National Research Ethics Service Committee North West Multi-Centre Haydock has approved the UKB cohort research and written informed consent to participate in the study was provided by all participants^14^. Approval for the use of this data was approved by the UK Biobank (project ID: 22881). The Harvard internal review board (IRB) deemed the research as non-human subjects research (IRB: IRB16-2145). Formal consent was obtained by the UK Biobank (https://biobank.ctsu.ox.ac.uk/ukb/ukb/docs/Consent.pdf).

### COVID-19 outcomes

In our investigation, we analyze two major COVID-19-related outcomes in UKB participants, COVID-19 test positivity, determined with microbiological (reverse transcriptase-polymerase chain reaction [RT-PCR]) testing^14^) and hospitalization due to COVID-19. We defined the outcome COVID-19 test positivity as the presence of at least one positive test result for a participant.

### Exposures prior to COVID-19 testing

We investigated the association of 360 “exposures” that included (a) clinical and diagnostic biomarkers of chronic disease and infection, (b) “environmental” factors, and (c) self-reported, doctor-diagnosed health and disease indicators with COVID-19 positivity and hospitalization. We use data measured during baseline visits 10–14 years (2006–2010) before the COVID-19 time periods.

The 63 real-valued biomarkers spanned five categories included adiposity and body characteristics (4 biomarkers, e.g., body mass index), blood count (23 biomarkers, e.g., white blood cell count), blood biochemistry (30 biomarkers, e.g., alkaline phosphatase), cardiovascular function (3 biomarkers, e.g., diastolic blood pressure), and lung function (3 biomarkers, e.g., forced vital capacity). Further, we use data measured during baseline visits (2006–2010). We performed rank-based inverse normal transformation (INT) to compare their associations across different models. We performed INT transformation using the RNOmni package (rankNorm function) with the offset parameter set to 0.5.^15^

Second, we investigated the association of 283 environmental factors in 14 categories (e.g., smoking, estimated nutrients consumed yesterday, infectious antigens) with COVID-19 positivity and hospitalization. We averaged the quantitative environmental factors (that fell under the estimated nutrients consumed yesterday [23 exposures] and infectious antigens [25 exposures] categories) over measurements from multiple instances or visits. For environmental factors that did not have many observations in subsequent instances, we used only the data from the baseline visit (first instance of measurement collected during 2006–2010) (e.g., environmental factors from the estimated nutrients yesterday category). We also performed INT-transformation of these factors (similar to the transformation of biomarkers) (as was also suggested by Millard et al.^16^). For categorical variables (which were also collected from multiple visits of a participant to the assessment center), we used data from the baseline visit (first instance of measurement collected during 2006–2010) as this contained the highest number of observations. Additionally, categorical variables with multiple levels were converted to sets of binary variables where each binary variable indicates whether a participant has a given value of this variable (as was suggested by Millard et al.)^16^. Ordinal categorical environmental factor variables were analyzed by treating such variables as continuous variables and real-valued quantitative environmental factor variables were scaled.

Third, we considered 14 health and disease indicators in 6 categories (“overall health rating”, “diabetes diagnosed by doctor”, “cancer diagnosed by doctor”, “vascular/heart problems diagnosed by doctor”, and “blood clot, DVT, bronchitis, emphysema, asthma, rhinitis, eczema, allergy diagnosed by doctor”) with COVID-19 positivity and hospitalization. Moreover, we considered baseline demographic variables in our analysis including sex, age, age squared, assessment center, ethnicity, average total household income after tax, and 40 genetic principal components. Similarly, for these variables we used data from the baseline visit (first instance of measurement collected during 2006–2010).

### Data-driven association to identify risk factors associated with future COVID-19 positivity and hospitalization

Niedzwiedz et al. report that Poisson regression may be preferred over logistic regression as odds ratios are often misinterpreted and Poisson regression allows for relative risks to be reported. As mentioned by highly cited COVID-19 papers (e.g.,^2^), using robust standard errors will help ensure “accurate estimation of *p*-values”. Zou shows error for estimated relative risk will be overestimated when Poisson regression is applied to binomial data. Therefore, Zou suggests robust standard errors may be an optimal solution to help overcome overestimation. We used Poisson regression (with log link) models with robust standard errors to associate each of the 360 factors and COVID-19 positivity (individually), while adjusting for sex, age, age squared, assessment center, ethnicity, average total household income after tax, and 40 genetic principal components (computed and provided by the UK Biobank). The model can be represented as log (π(x_i_)) = Exposure + Age + Age + Sex + Assessment Center + Income + Genetic Principal Component 1 + Genetic Principal Component 2 + … + Genetic Principal Component 40 + log(t_i_) where we assume that subject i has an underlying risk for a COVID-19 related outcome that is a function of x_i_, as π(x_i_). As Zou mentions, “since π(x_i_) must be positive, the logarithm link function is a natural choice for modeling π(x_i_)”^17^ and log(t_i_) is the offset of time in years between date of baseline visit and date of first positive COVID-19 test^17^.

The approach of using robust standard errors involves correcting the original model-based standard errors using the variation of the difference between observed outcome values and predicted values from the model (or the residuals)^17,18^ Moreover, Mansournia et al. note the reason why this approach is also referred to as sandwich estimation^18^. Mansournia et al. mention that the terms corresponding to the variance based on the residuals is “sandwiched” in between the terms corresponding to the variance based on the model^18^. For details on the mathematical derivation of the approach, please see Zou et al.^17^.We report risk ratios and adjusted corresponding *p*-values for multiple comparisons using the false discovery rate (FDR) approach^19^. Also, we perform a sensitivity analysis running logistic regression and estimating odds ratios for COVID-19 positivity across both timepoints.

Additionally, testing strategy during the time periods considered may confound the associations we observe. Given that there were two major phases of the testing strategy (Pillar I and Pillar II) where the first phase prioritized individuals with health complications and healthcare workers, it was unclear which tests performed on UK Biobank participants corresponded to Pillar II versus Pillar I; therefore, we ran a sensitivity analysis to adjust for criteria that were used to prioritize individuals to be tested—healthcare workers and people with health complications. Moreover, it has been shown by Williamson et al.^20^ that health complications most associated with COVID-19-related death earlier in the pandemic include obesity and diabetes. In order to account for testing strategy-related confounding effects, we additionally adjust for healthcare worker status, BMI (body mass index), diabetes, haematological malignancies (lymphoma, leukemia, multiple myeloma, myelofibrosis or myelodysplasia, and other haematological malignancy) and usage of immunosuppressants (see Supplementary Table 2 for medication codes used to identify immunosuppressants^21^) in addition to the baseline demographic covariates in the aforementioned analysis.

Similarly, we investigated the association of all 360 risk factors with COVID-19 hospitalization.

Also, we sought to quantify the difference in exposure associations with COVID-19 positivity between time points. We executed a Poisson regression (with log link) models for individual exposures (as described above) with an additional interaction term between each exposure and the time point variable (codified as a dummy variable to indicate the first [tests until July 17, 2020] or second time point [tests between July 18, 2020 and February 2, 2021]). We also report risk ratios for each interaction term and adjusted corresponding *p*-values for multiple comparisons using the false discovery rate (FDR) approach^19^.

### Probing the variation of associations due to model choice via “vibration of effects”

Next, we executed a large sensitivity analysis to examine the fragility of associations due to model and covariate choice, in a large sensitivity analysis called the vibration of effects (VoE)^11,22^ for the top 12 exposures (ascertained by FDR values) identified from our analysis. Through our data-driven exposure-wide approach, we identified 12 FDR-significant exposures (FDR-corrected *p*-value in top 10 percentile) in the initial time period, including 1) “Apolipoprotein A”, 2) “Own accommodation outright”, 3) “Nitrogen oxides air pollution; 2010”, 4) “Current frequency of shift work”, 5) “Townsend deprivation index at recruitment”, “Body mass index (BMI)”, “HDL cholesterol”, “Urban (less sparse) home area population density”, “Qualifications (no education)”, “Son and/or daughter (including step-children) in household”,“Exposure to tobacco smoke outside home”, and “Alcohol intake frequency”. Given the computational complexity of running all possible models for all significant exposures, we selected the 12 exposures that included ones that were prominently featured (FDR in top 10 percentile) in our analysis) and mostly did not include multiple exposures from the same category (for example we did not include “nitrogen dioxide air pollution; 2010”). The set of varying adjustments in the models included the 12 exposures and the “baseline” variables that we kept in all models were gender, age, age squared, assessment center, ethnicity, and average total household income after tax. We performed the VoE analysis in the context of the 12 exposures as varying adjustments by running models with all possible combinations of adjustments from the set of 12 exposures. We ran a total of 8192 (2^12^) models, while keeping the sample size (*n* = 2821) the same for all (as it has been suggested previously^4^)). We used Poisson regression models (with log link) to associate variables with COVID-19 positivity and extracted beta-estimates to compute risk ratios (RR). The heuristic that we used for computing VoE was as defined by Patel et al.^4^.

### Statistics and reproducibility

All data processing and subsequent analyses were done using R Version 3.6.1 on O2 which is a high-performance computing cluster at Harvard Medical School that runs on Linux. We made our code accessible at (https://github.com/stejat98/UKB_COVID_XWAS) and on Zenodo (10.5281/zenodo.7542752)^23^. Summary statistics (including risk ratios, FDR-corrected *p*-values, sample sizes, etc.) can be found in Supplementary Data 1–10.

### Reporting summary

Further information on research design is available in the Nature Portfolio Reporting Summary linked to this article.

## Results

### Baseline characteristics

The baseline characteristics of the UK Biobank (UKB) cohort participants are reported in Supplementary Data 1. Out of 9268 individuals tested in the UKB cohort for COVID-19 until July 17, 2020, results from 1544 patients (16.7%) were positive. Out of the 38,837 participants tested in the UKB cohort for COVID-19 between July 18, 2020 and February 2, 2021, results from 8539 patients (22%) were positive. Of the 1544 patients that tested positive until July 17, 2020, 1011 (65.5%) were hospitalized and 243 (15.7%) died (Supplementary Data 2). Overall, age was significantly associated with COVID-19 positivity (Risk Ratio [RR] for a 10 year increase in age: 0.807, 95% CI: [0.757, 0.86], FDR: 1.03 × 10^−9^), in addition to geographic proxy variables (assessment centers) such as Glasgow (7.91, [4.62,13.5], 1.78 × 10^−12^) among cases tested until July 17, 2020 [Table 1].

**Table 1:** Top baseline demographic associations for COVID-19 positivity for first time point (tests until 07/17/2020).

	COVID-19 positivity			COVID-19 hospitalization		
Baseline demographic variable	RR (95% CI)	*p*-value	FDR	RR (95% CI)	*p*-value	FDR
Glasgow	7.91 (4.62–13.5)	4.75E−14	1.78E−12	1.13E−05 (1.58E−06 to 8.10E−05)	7.92E−30	5.94E−28
Age	0.807 (0.757–0.860)	4.11E−11	1.03E−09	1.09 (1.07–1.11)	8.02E−24	1.50E−22
Leeds	3.05 (1.99–4.69)	3.33E−07	6.24E−06	1.86 (1.59–2.17)	4.78E−15	3.58E−14
Liverpool	2.70 (1.75–4.16)	6.60E−06	9.90E−05	1.98 (1.70–2.31)	2.90E−18	3.10E−17
Sheffield	2.63 (1.71–4.07)	1.24E−05	1.55E−04	1.44 (1.21–1.70)	2.46E−05	0.000108
Gender (Male)	1.21 (1.09–1.34)	2.75E−04	2.95E−03	1.06 (1.04–1.09)	1.00E−06	4.71E−06
Average total household income before tax (<18,000 Euros)	1.72 (1.26–2.33)	5.51E−04	5.16E−03	1.02 (0.947–1.10)	0.59	0.835
Middlesborough	2.06 (1.31–3.24)	1.67E−03	1.39E−02	2.26 (1.94–2.62)	2.83E−26	1.06E−24
Croydon	1.95 (1.25–3.04)	3.13E−03	2.13E−02	1.34 (1.12–1.59)	1.13E−03	4.71E−03
Average total household income before tax (31,000 to 51,999 Euros)	1.59 (1.17–2.15)	3.05E−03	2.13E−02	1.02 (0.947–1.10)	0.585	0.835

Of the 8539 patients that tested positive until February 2, 2021, 2150 (25.2%) were hospitalized and 169 (1.98%) died (Supplementary Data 2). Similar to the results from the previous time point, age was significantly associated with COVID-19 positivity (Risk Ratio [RR] for a 10 year increase in age: 0.595, 95% CI: [0.581, 0.610], FDR < 1 × 10^−64^), in addition to geographic proxy variables (assessment centers) such as Cardiff (6.37, [5.15, 7.88], 7.99 × 10^−64^) and Swansea (10.5, [8.03, 13.8], 6.33 × 10^−64^) among cases tested until February 2, 2021 [Supplementary Data 3].

### Data-driven identification of risk factors associated with future COVID-19 positivity

**Fig. 1:**
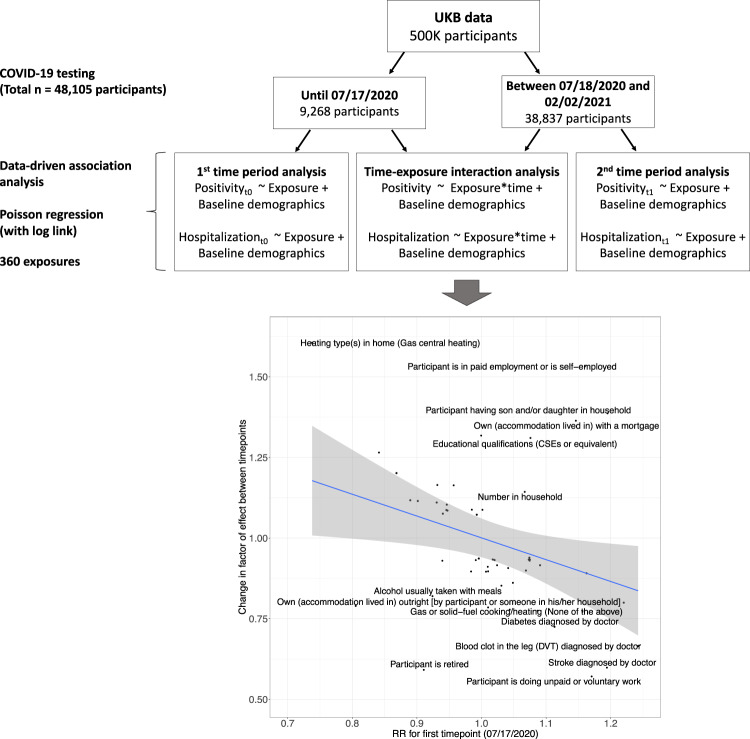
Schematic overview of data-driven analysis of COVID-19 positivity risk factors across two timepoints. This schematic diagram depicts the analytic pipeline. (1) COVID-19 testing data was collected from two time periods (until 07/17/2020 and between 07/18/2020 and 02/02/2021). (2) Data-driven association analysis was performed for each of the 360 exposures using Poisson regression (with log link). Associations were computed for each time period separately. Additionally, models were run to assess time-exposure interaction effects. The blue line on the scatterplot represents a linear regression line and the grey shading around it represents the 95% confidence interval.

**Fig. 2:**
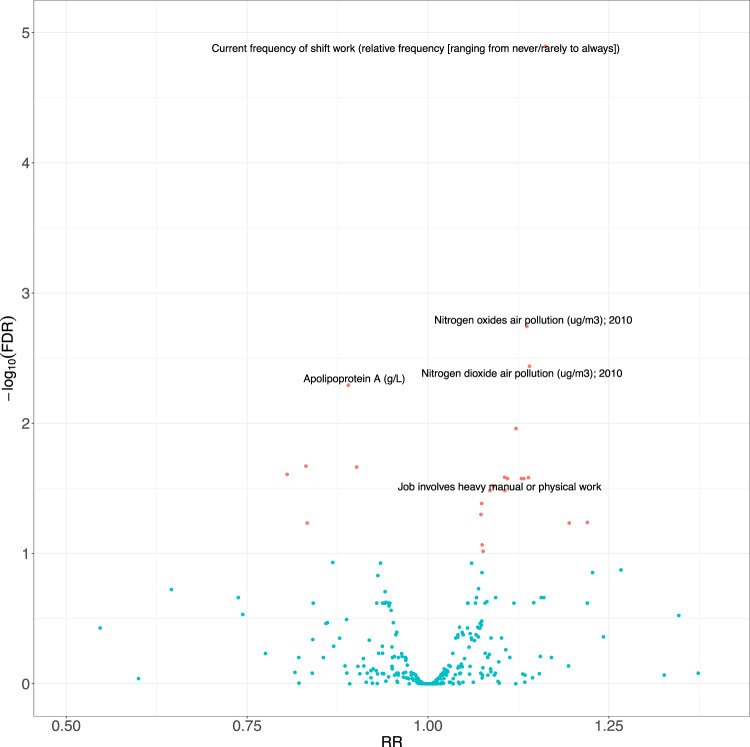
Association size versus -log10(adjusted *p*-values) between 360 exposures and COVID-19 positivity for first time point (tests until 07/17/2020). The risk ratios (RR) versus the negative log (base 10) of FDR-corrected *p*-values for 360 different exposures. The red color indicates FDR < 0.1 significant exposures and the blue color indicates FDR > 0.1 exposures. For the underlying dataset, *n* = 9268.

**Fig. 3:**
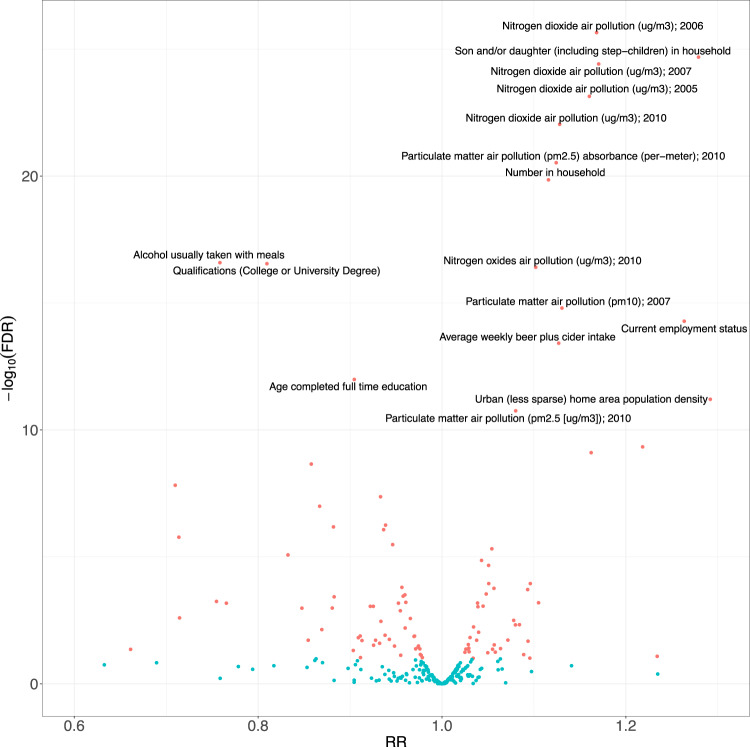
Association size versus -log10(adjusted *p*-values) for COVID-19 positivity for second time point (tests between 07/18/2020 and 02/02/21). The risk ratios (RR) versus the negative log (base 10) of FDR-corrected *p*-values for 360 different exposures. The red color indicates FDR < 0.1 significant exposures and the blue color indicates FDR > 0.1 exposures. For the underlying dataset, *n* = 38,837.

**Fig. 4:**
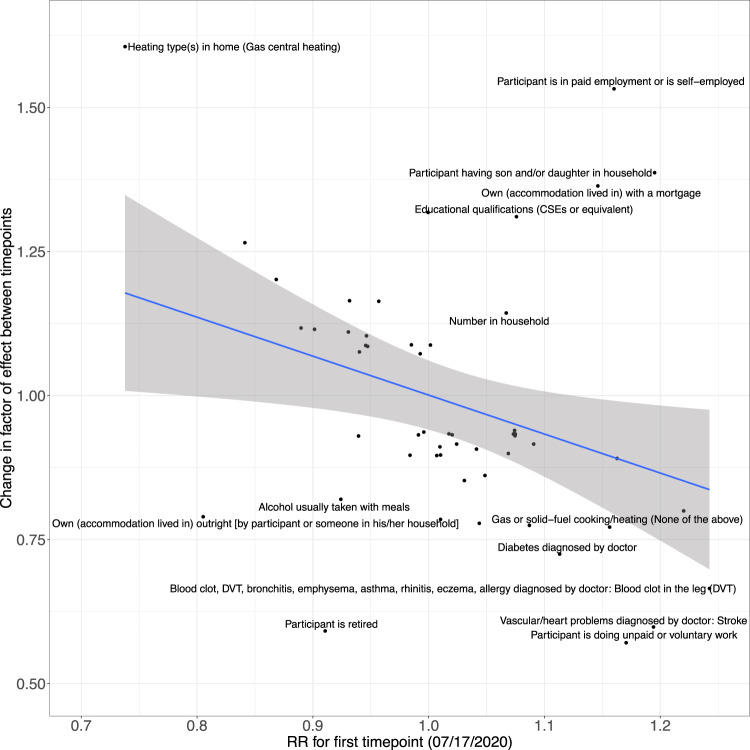
Change in relative risk between participants sampled in 2/02/2021 and 7/17/2020 vs. relative risk for participants sampled in July 2020 (x-axis). Scatterplot of associations with FDR < 0.1 significant interaction effects between risk ratios (RR) for first time point (07/17/2020) and change in factor of effect between time points. Exposures that significantly deviate from the overall negative linear trend are labelled. The blue line on the scatterplot represents a linear regression line and the grey shading around it represents the 95% confidence interval.

We systematically associated environmental factors, biomarkers, and health indicators with COVID-19 positivity for two different samples during two different timepoints: before and inclusive of July 17, 2020 and between July 18, 2020 and February 2, 2021 (Fig. 1). We identified 31 significant exposures (27 environmental factors and 4 biomarkers) (Fig. 2, Supplementary Data 4) and 36 significant exposures (34 environmental factors and 2 biomarkers and health indicators) (Fig. 3, Supplementary Data 5) that had FDRs in the top 10 percentile of all associations tested with thresholds of FDR-corrected *p*-value of less than 0.141 and FDR-corrected *p*-value of less than 1.94 × 10^−4^ respectively at each time (Supplementary Figs. 1, 2). It is also important to note that while we tested the same number of exposures (360) for both timepoints we had low sample sizes for 45 exposures that were all infectious antigens, leaving 315 exposures to report results for this first time point. As exposures associated with COVID-19 positivity varied between time points, we systematically quantified the difference in relative risk ratios of exposures between the two samples by testing for the interaction between each exposure variable and the time point variable and reported interaction term risk ratios. We identified 35 exposures with a FDR-corrected *p*-value in the top 10 percentile across all 360 exposures tested for the interaction effect with time point (Supplementary Data 6). The interquartile range of the interaction term Risk Ratios is 0.857 and 1.31. We also observed an overall negative trend between the difference in RRs and the RRs for the first timepoint (07/17/2020) in Fig. 4, thereby indicating an overall decrease in the association sizes between the two samples.

We begin by describing exposures found in each sample separately. For individuals sampled before July 17, 2020, the top significantly associated factor (in terms of FDR-significance) was “current frequency of shift work” (e.g.^24^) (RR [for a 1 standard deviation (SD) change in relative frequency of shift work]: 1.16, 95% CI: [1.10, 1.23], FDR: 8.20 × 10^−6^, ΔAUC [Full Model AUC - AUC of baseline demographic covariates-only model]: 8.79 × 10^−3^ [0.679–0.67]) (Supplementary Table 1 for distribution of COVID-19 prevalence across shift work categories). For individuals who report they always conduct shift work, the prevalence of COVID-19 positivity was 28.6% and for individuals who report that they never conduct shift work, the prevalence of COVID-19 positivity was almost half that reported always, 15.2%.

However, and on the other hand, we did not find “current frequency of shift work” to be significantly associated with cases tested between July 18, 2020 to February 2, 2021 (RR [for a 1 standard deviation (SD) change in relative frequency of shift work]: 1.01, 95% CI: [0.989, 1.03], FDR: 0.564). The interaction term RR was 0.891 (FDR: 6.80 × 10^−4^), which corresponded to a significant decrease by a factor of 10.9% as time progressed between the two samples.

We found that the “current frequency of shift work” is associated with positivity in the early time period. However, the association could be influenced by the type of worker, such as a healthcare worker. We tested the association of current frequency of shift work in the healthcare worker (HCW) sample and compared it to the association in non-HCW sample. We found the associations to be similar between non-HCW (RR: 1.11, 95% CI: [1.04, 1.18], *p*-value: 2.63 × 10^−3^) and HCW-only (RR: 1.18, 95% CI: [1.05, 1.32], *p*-value: 3.79 × 10^−3^) samples, indicating that being a healthcare worker is independent of the association between frequency of shift work and COVID-19 positivity.

Other top factors include “nitrogen oxides air pollution measured in 2010” (RR: 1.14 [for a 1 SD change in nitrogen oxide air pollution], 95% CI: [1.08, 1.21], FDR: 4.16 × 10^−4^, ΔAUC: −2.18 × 10^−4^ [0.648–0.649] and “nitrogen dioxide air pollution also measured in 2010” (RR [for a 1 SD change]: 1.15, 95% CI: [1.08, 1.23], FDR: 6.88 × 10^−4^, ΔAUC: −1.29 × 10^−3^ [0.647–0.649]). For cases up to February 2, 2021, we found “nitrogen oxides air pollution measured in 2010” (RR: 1.10 [for a 1 SD change in nitrogen oxide air pollution], 95% CI: [1.08, 1.13], FDR: 3.89 × 10^−17^) and “nitrogen dioxide air pollution measured in 2010” (RR: 1.13 [for a 1 SD change], 95% CI: [1.1, 1.15], FDR: 9.11 × 10^−23^) to be significant. The interaction term RRs were 0.985 (FDR: 0.763; decrease by a factor of 1.5% between timepoints) and 1.00 (FDR: 0.959; no change between timepoints), respectively. In total, we identified 11 exposures and 9 exposures to be significant in the air pollution category of exposures for cases leading up to July 17, 2020 and February 2, 2021, respectively.

The top two significantly associated biomarkers with COVID-19 positivity were apolipoprotein A (RR [for a 1 SD change that is equivalent to 0.272 g/L]: 0.889, 95% CI: [0.839, 0.942], FDR: 3.25 × 10^−3^, ΔAUC: −5.30 × 10^−4^ [0.646–0.646]) and HDL cholesterol (RR [for a 1 SD change that is equivalent to 0.382 mmol/L]: 0.900, 95% CI: [0.849, 0.955], FDR: 1.11 × 10^−2^, ΔAUC: −1.08 × 10^−3^ [0.644–0.645]). HDL cholesterol (RR: 0.995, 95% CI: [0.972, 1.02], FDR: 0.785, ΔAUC: −1.08 × 10^−3^ [0.644–0.645]) was also implicated in cases between July 18 and February 2, 2021, but apolipoprotein A (RR: 1.00, 95% CI: [0.978, 1.02], FDR: 0.978) was not. The interaction term RRs for HDL cholesterol and apolipoprotein A were 1.11 (FDR: 3.99 × 10^−3^; significant increase by factor of 11% between samples) and 1.12 (FDR: 3.01 × 10^−3^; significant increase by factor of 12% between samples), respectively. The interquartile range (IQR) of the number of complete cases across all associations for individuals sampled before July 17,2020 is [4860, 7326]. We also plot the sample size versus risk ratio of COVID-19 positivity associations (Supplementary Fig. 3).

For cases between July 18, 2020 and February 2, 2021, the top associations included “nitrogen dioxide air pollution measured in 2006” (RR: 1.17 [for a 1 SD change], 95% CI: [1.14, 1.2], FDR: 2.23 × 10^−26^, ΔAUC: 6.71 × 10^−3^ [0.727–0.72]), “participant having son and/or daughter in household” (RR: 1.28, 95% CI: [1.22, 1.34], FDR: 2.06 × 10^−25^, ΔAUC: 6.21 × 10^−3^ [0.726–0.72]), and “number of people in participant’s household” (RR: 1.12 [for a 1 SD change], 95% CI: [1.09, 1.14], FDR: 1.41 × 10^−20^, ΔAUC: 5.78 × 10^−3^ [0.726–0.72]). For cases up to July 17, 2020, “participant having son and/or daughter in household” (RR: 1.19, 95% CI: [1.06, 1.35], FDR: 0.06) and “nitrogen dioxide air pollution measured in 2006” (RR: 1.14 [for a 1 SD change], 95% CI: [1.06, 1.23], FDR: 0.01) was significant but “number of people in participant’s household” (RR: 1.07 [for a 1 SD change], 95% CI: [1.01, 1.13], FDR: 2.06 × 10^−1^) was not significantly associated with COVID-19 test positivity. The interaction RRs for “participant having son and/or daughter in household”, “nitrogen dioxide air pollution measured in 2006”, and “number of people in participant’s household” are 1.39 (FDR: 3.04 × 10^−7^; significant increase by factor of 39% between timepoints), 1.02 (FDR: 0.659; non-significant increase by factor of 2% between timepoints), and 1.14 (FDR: 9.18 × 10^−6^; significant increase by factor of 14% between timepoints), respectively. In particular, “participant having son and/or daughter in household” and “number of people in participant’s household” are notable exceptions to the overall decreasing trend in the association sizes between the two samples. The interquartile range (IQR) of the number of complete cases across all associations for individuals sampled between July 18, 2020 and February 2, 2021 is [16,183, 31,521]. We also plot the sample size versus risk ratio of COVID-19 positivity associations (Supplementary Fig. 4).

### Assessing robustness of identified COVID-19 positivity associations to testing strategy

Additionally, testing strategy (prioritization of health care workers and/or individuals with chronic disease) during the time periods considered may confound the associations we observe. In order to account for testing strategy-related confounding effects, we additionally adjust for healthcare worker status, BMI (body mass index), diabetes, haematological malignancies and usage of immune suppressants, in addition to the baseline demographic covariates in the aforementioned analysis. We report associations after adjusting for testing strategy-related confounding effects and contextualize these with the estimates not adjusted for testing strategy-related effects that we reported in the previous section. For individuals sampled before July 17, 2020, “current shift frequency”: (RR: 1.12, 95% CI: [1.06, 1.19], FDR: 1.22 × 10^−2^) [testing strategy effects-unadjusted: RR: 1.16, 95% CI: (1.10, 1.23), FDR: 8.20 × 10^−6^], “nitrogen oxides air pollution measured in 2010” (RR: 1.13, 95% CI: [1.06, 1.19], FDR: 1.22 × 10^−2^) [testing strategy effects-unadjusted: RR: 1.14, 95% CI: [1.08, 1.21], FDR: 4.16 × 10^−4^] and “nitrogen dioxide air pollution measured in 2010” (RR: 1.13, 95% CI: [1.06, 1.20], FDR: 1.77 × 10^−2^) [testing strategy effects-unadjusted: RR: 1.15, 95% CI: [1.08, 1.23], FDR: 6.88 × 10^−4^], remained among the top associations and were concordant in direction with associations reported prior to adjustment of the additional covariates. Similarly, for individuals sampled between July 18, 2020 and February 2, 2021, “participant having son and/or daughter in household” (RR: 1.28, 95% CI: [1.22, 1.34], FDR: 7.92 × 10^−25^) [testing strategy effects-unadjusted: RR: 1.28, 95% CI: (1.22, 1.34), FDR: 2.06 × 10^−25^] and “number of people in participant’s household” (RR: 1.11, 95% CI: [1.09, 1.14], FDR: 9.81 × 10^−20^) [testing strategy effects-unadjusted: RR: 1.12, 95% CI: (1.09, 1.14), FDR: 1.41 × 10^−20^] remained among the top associations and were concordant in direction with associations reported prior to adjustment of the additional covariates.

### Comparing model selection robustness across critical points in the pandemic in association strength and size

We compared the variation due to time of sampling versus the variation due to covariate choice (Vibration of Effects [VoE]). Specifically, to test whether the associations differ due to model choice and compare them with the main findings of our study, we estimated the vibration of effects (VoE)^11,22,25^ for 12 variables that were initially identified as significant (FDR < 0.1) from our analysis (for cases up to July 17, 2020) at both timepoints (see Methods). For 7 out 12 FDR significant exposures, the time of sampling exhibited greater variation in RR versus model selection as detected by VoE. For example, apolipoprotein A, had a range of [0.572, 0.713] in cases up to July 17, 2020 and a range of [1.00, 1.05] in cases up to February 2, 2021. “Son and/or daughter in household” had ranges of [1.07, 1.12] and [1.24, 1.26] in cases up to July 17, 2020 and February 2, 2021, respectively. Patel et al. and Tierney et al.^11,25^ have shown that empirically estimating the VoE makes it possible to detect significant differences in exposure associations can arise just due to choice of covariate (which can lead to multiple modes of associations otherwise referred to as the “multimodality of effects”^11^). To illustrate this, we plotted the vibration of effects for “current frequency of shift work”, “nitrogen oxides air pollution”, and BMI for cases up to July 17, 2020 (Supplementary Fig. 5) and February 2, 2021 (Supplementary Fig. 6).

For example, “current frequency of shift work” did not display multimodality of effects as associations were consistent (had the same size) across all adjustment scenarios. Among participants tested up to July 17, 2020, all associations were in the positive direction and lying between 1.11 and 1.14 (Supplementary Fig. 5a). However, for participants tested between July 18, 2020 and February 2, 2021, “current frequency of shift work” were not consistent overall as the range of associations (1.00 and 1.01) included 1, thereby suggesting a null association (Supplementary Fig. 6a). Also risk ratios and -log_10_(*p*-values) shrank from the previous time point.

The overall VoE for nitrogen oxides air pollution indicates positive association with COVID test positivity (RRR: 1.04, RP: 0.732). We identified four different modes. We visualized VoE by coloring the points based on the inclusion/exclusion of the 12 other significant exposures (providing 12 separate visualizations). For nitrogen oxide air pollution, we found that the four modes were indicative of the presence (or absence) of the Townsend deprivation index and Urban (less sparse) home area population density variables in the models (Supplementary Fig. 6b). The models containing Townsend deprivation index had smaller RR and smaller -log_10_(*p*-value) for nitrogen oxide air pollution. On the other hand, models containing Urban (less sparse) home area population density had smaller -log_10_(*p*-value) for nitrogen oxide air pollution.

We also identified four modes in the associations between BMI and COVID-19 positivity (RRR: 1.01, RP: 0.925). The VoE plots (Supplementary Figs. 5c, 6c) indicated that the multimodality of BMI associations was driven by the presence (or absence) of apolipoprotein A and HDL cholesterol in the models. For instance, the -log_10_(*p*-value) for BMI risk ratio decreases with HDL cholesterol in the models. For participants tested between July 18, 2020 and February 2, 2021, we again found multiple modes that were indicative of the presence (or absence) of the Townsend deprivation index and Urban (less sparse) home area population density variables in the models associating nitrogen oxide air pollution (RRR: 1.02, RP: 3.06) with COVID-19 positivity. However, while the inclusion of the Townsend deprivation index (an area level/local geographic indicator of socioeconomic status) and Urban (less sparse) home area population density variable caused the risk ratios and -log_10_(*p*-values) to shrink, nitrogen oxide air pollution associations from all model combinations still attained significance. Similarly, despite the shrinkage in -log_10_(*p*-values) due to inclusion of apolipoprotein A and HDL cholesterol in the models, BMI associations from all possible model combinations (RRR: 1.00, RP: 2.95) still were significant. Overall, we note the tension between decreasing risk ratios and increasing -log_10_(*p*-values) for exposure associations with test positivity as the number of tests (and cases) also increase.

### Significant factors identified for COVID-19 hospitalization

In addition, we systematically associated all factors with COVID-19 hospitalization (Supplementary Data 7) for cases up to July 17, 2020. We identified 28 of these factors to meet a threshold of FDR-corrected *p*-value in the top 10 percentile of associations tested (FDR-corrected *p*-value equivalent of 0.29 in top 10%). Given the high *p*-values observed across associations for this time point we also report the FDR-corrected *p*-value equivalent for the top 1% of associations tested to be 0.0121. Top factors included alanine aminotransferase (RR [for a 1 SD change that is equivalent to 14.5 U/L]: 1.03, 95% CI: [1.02, 1.04], FDR: 3.85 × 10^−3^, ΔAUC: 1.84 × 10^−4^ [0.732–0.732]) and glycated haemoglobin (HbA1c) (RR [for a 1 SD change that is equivalent to 8.29 mmol/mol]: 1.03, 95% CI: [1.01, 1.04], FDR: 6.34 × 10^−3^, ΔAUC: 1.66 × 10^−3^ [0.731–0.729]).

Similarly, for cases up to February 2, 2021, we systematically associated all factors with COVID-19 hospitalization (Supplementary Data 8). We identified 32 of those factors to meet a threshold of FDR-corrected *p*-value in the top 10 percentile of associations tested (FDR-corrected *p*-value threshold of 2.93 × 10^−9^). Top factors for COVID-19 hospitalization included overall health rating (RR: 1.06, 95% CI: [1.05, 1.07], FDR: 2.67 × 10^−37^, ΔAUC: −3.33 × 10^−3^ [0.683–0.686]) and unable to work because of sickness or disability (RR: 1.19, 95% CI: [1.15, 1.23], FDR: 4.32 × 10^−23^, ΔAUC: −5.27 × 10^−3^ [0.680–0.686]).

### Sensitivity analysis using logistic regression to estimate odds ratios for COVID-19 positivity during both timepoints

Additionally, we used logistic regression to compute odds ratios (OR) for COVID-19 positivity across both timepoints as a sensitivity analysis and compare the OR of top findings with the RR computed from Poisson regression models (with robust standard errors). In brief, we did not have evidence to suggest that use of the Poisson biased the associations we identified.

For individuals sampled before July 17, 2020, “current shift frequency”: (OR: 1.23, 95% CI: [1.13, 1.32], FDR: 1.14 × 10^−4^) [Poisson regression model with robust errors: RR: 1.16, 95% CI: (1.10, 1.23), FDR: 8.20 × 10^−6^], “nitrogen oxides air pollution measured in 2010” (OR: 1.19, 95% CI: [1.10, 1.28], FDR: 1.04 × 10^−3^) [Poisson regression model with robust errors: RR: 1.14, 95% CI: [1.08, 1.21], FDR: 4.16 × 10^−4^] and “nitrogen dioxide air pollution measured in 2010” (RR: 1.2, 95% CI: [1.1, 1.29], FDR: 1.16 × 10^−3^) [Poisson regression model with robust errors: RR: 1.15, 95% CI: [1.08, 1.23], FDR: 6.88 × 10^−4^], remained among the top associations and were concordant in direction with associations reported from Poisson regression model with robust errors (as we observed with our testing strategy effects sensitivity analysis). Similarly, for individuals sampled between July 18, 2020 and February 2, 2021, “participant having son and/or daughter in household” (RR: 1.44, 95% CI: [1.35, 1.54], FDR: 1.73 × 10^−6^) [Poisson regression model with robust errors: RR: 1.28, 95% CI: (1.22, 1.34), FDR: 2.06 × 10^−25^] and “number of people in participant’s household” (RR: 1.17, 95% CI: [1.14, 1.21], FDR: 9.73 × 10^−22^) [Poisson regression model with robust errors: RR: 1.12, 95% CI: (1.09, 1.14), FDR: 1.41 × 10^−20^] remained among the top associations and were concordant in direction with associations reported from Poisson regression model with robust errors (as we observed with our testing strategy effects sensitivity analysis).

## Discussion

As is expected, the type, size, and number of risk factors changes as the pandemic progresses. The thought that risk factors may evolve throughout the pandemic has in fact been suggested by others. For example, Roso-Llorach et al. assessed the differences in mortality and clinical variables and found socioeconomic disparities to increase as the pandemic progressed^3^. However, identifying how risk factors change has been elusive despite the explosion in risk models for COVID-19. To mitigate these challenges, we tested a comprehensive list of all risk factors simultaneously, while accounting for multiple hypotheses in two time points and found 31 early in the pandemic, with occupation-related factors being prominently featured, and 36 later in the pandemic. The number and diversity of risk factors found increased as the pandemic progressed, reflecting the increasing number of individuals testing positive.

We conclude that time of sampling has had an influence on association size and the number of variables identified with robust support. Given the sample sizes of participants for each time point (9268 and 38,837) and the number of significant exposures identified for each time point, more generally, we postulate that it may take a sample of 10,000 participants to see environmental sources of health disparity emerge from a convenience sample such as UK Biobank for as low as 10–20% increased/decreased risk. We also note an overall negative trend between the difference in RRs and the RRs for the first timepoint (07/17/2020), suggesting an overall decrease and “regression to the mean” in the association sizes between the two timepoints.

Specifically, early in the pandemic, we have identified employment type—specifically surrounding the frequency of shift work—and re-identified air pollution, particularly nitrogen oxide and dioxide as risk factors most significantly associated with COVID-19 positivity and hospitalization, albeit with modest RR. Additionally, we found the association of “current frequency of shift work” to be robust to the healthcare worker (HCW) and non-HCW status sampled before July 17, 2020. Furthermore, while “current frequency of shift work” has a positive association with COVID-19 positivity, it has a negative association with COVID-19 hospitalization. We hypothesize that this opposite risk could potentially be indicative of socioeconomic barriers to hospital treatment, despite testing being made relatively accessible across socioeconomic strata. Moreover, we report negative associations (with hospitalization) of baseline demographic factors such as “Average total household income before tax (Less than 18,000 Euros)” [RR: 0.36, 95% CI: (0.16, 0.83)] and “Average total household income before tax (31,000 to 51,999 Euros)” [RR: 0.36, 95% CI: (0.15, 0.83)]. However, it is important to note that we found the association of “current frequency of shift work” to be negative after adjusting for the baseline demographic factors, including income. This suggests that individuals, who have jobs that involve a higher shift frequency are less likely to be admitted to the hospital early on in the pandemic. We suspect that participants who have a job with a higher frequency of shifts tend to not admit themselves to a hospital, potentially out of fear of job loss or other disciplinary proceedings.^26^ We also note that we do not find this factor to be significantly associated with COVID-19 positivity later in the pandemic (cases between July 18th 2020 and February 2nd 2021).

It is also important to consider age, whose importance as a risk factor for COVID-19 outcomes/complications has been widely reported in the literature. Notably, we report that the RR for age (per 10 years) is less than 1 for COVID-19 positivity and is greater than 1 for COVID-19 hospitalization as we show in Table 1 and Supplementary Data 3. For example, for the first time point age had a RR of 0.807 for COVID-19 positivity and RR of 1.18 for COVID-19 hospitalization and for the second time point age had a RR of 0.595 and for COVID-19 positivity and a RR of 2.63 for COVID-19 hospitalization. These estimates are concordant in direction with age RR estimates for COVID-19 positivity and hospitalization reported elsewhere^1^.

Later in the pandemic, we found household factors, such as “participant having son and/or daughter in household” and “number of people in participant’s household” associated with COVID-19 positivity and also found these associations to be robust to testing strategy criteria. Specifically, “participant having son and/or daughter in household” had a larger risk ratio than earlier in the pandemic (with an interaction term RR of 1.39 [FDR: 3.04 × 10^−7^; significant increase by factor of 39% between timepoints]). Similarly, “number of people in participant’s household” had a larger risk ratio and smaller FDR-corrected *p*-value later in the pandemic than earlier in the pandemic (with an interaction RR of 1.14 [FDR: 9.18 × 10^−6^; significant increase by factor of 14% between timepoints]). Both variables are notable exceptions to the overall negative trend between the difference in RRs and the RRs for the first timepoint (07/17/2020) in Fig. 3. Also, it is essential to consider that the majority of UKB participants are senior citizens of retired age (most participants were 60–69 years old)^27^ and younger individuals may have been going out more for work as the pandemic progressed. Thus, we suspect that having a son or daughter or a greater number of people in the household may have increased the chance of such younger individuals bringing the virus in from outside of the household.

It is important to evaluate the clinical significance of household factor associations for COVID-19 positivity with respect to established risk factors in the literature (e.g., age) as the pandemic progressed. For example, “participants having a son and/or daughter in their household” accounted for an increase in incidence from 20% to 32% (incidence risk difference of 12%) between timepoints. On the other hand, for elderly participants (age >65) incidence decreased from 16% to 13% (difference of −3%).

It is also important to evaluate the relevance of geographic proxy variable risk factors such as assessment centers. Such geographic proxy factors may help serve as indicators of socioeconomic differences between groups. We report the proportion of participants in each family income category for each assessment center in Supplementary Data 9 and 10. For example, we identified during the second time period (tests between July 18, 2020 and February 2, 2021) the Swansea assessment center as the one that confers the greatest risk for testing positive (RR [95% CI]: 10.5, [8.03, 13.8], FDR: 6.33 × 10^−64^ vs. Oxford). Swansea has 27.9% of its tested participants belonging to the lowest family income category (less than 18,000 Euros). In comparison, the reference location, Oxford, had 16.8%.

We also report that many associations are influenced by modeling assumptions; however, time of sampling seems to exhibit a greater variation in associations versus model selection as detected by VoE. For example, while the association risk ratios for “current shift frequency” for the first timepoint (cases up to July 17th 2020) ranged between 1.11 and 1.14 (variation by a factor about 3% due to presence or absence of factors in the models) in “current frequency of shift work”, the interaction term (between exposure and time point) RR was 0.891 (FDR: 6.80 × 10^−4^), which corresponded to a significant decrease by a factor of 10.9% as time progressed between the two samples, thereby rendering the association null later in the pandemic. This seems to provide further evidence for the suggestion that COVID-19 observational associations are dependent on the time of pandemic.

The COVID-19 Host Genetics Initiative conducted a genome-wide association study (GWAS) and a subsequent meta-analysis across cohorts (using study-specific summary statistics) to identify genetic risk factors associated with COVID-19^28^. Non-genetic risk factors with COVID-19 outcomes compare with those of genetic risk factors. Here, we focus on explicitly comparing genetic and nongenetic risk factor associations with COVID-19 hospitalization. For example, the COVID-19 Host Genetics Initiative reported an odds ratio (OR) for rs2271616’s association with COVID-19 hospitalization to be OR [95% CI] = 1.12 [1.06, 1.19]. We report here that nongenetic risk factors whose associations with COVID-19 hospitalization are comparable in magnitude with that of the OR of rs2271616. Other teams have executed analyses varying in consistency and breadth of candidate subsets of risk factors at a time in the UKB. For example, Niedzwiedz et al. specifically assessed the association of ethnicity with COVID-19 susceptibility and found certain minority ethnic groups to be associated with greater risk after adjusting for socioeconomic differences and behavioral risk factors or baseline health^2^. Hastie et al. examined only one biomarker, vitamin D, and analyzed its association with risk of COVID-19 positivity and ethnic differences in COVID-19 positivity and found no link^29^. However, others have shown conflicting results: an association between Vitamin D deficiency and hospitalization but no causal relationship between Vitamin D levels and COVID-19 severity^30^.

In contrast, much can be learned when moving beyond isolated sets of candidate factors. For example, Chadeau-Hyam et al. examined subsets of factors across multiple categories (social, environmental, demographic factors) and pressure-tested a suite of sensitivity analyses^1^. Here, we examine a wider range of risk factors across categories across two timepoints and compare 63 laboratory biomarkers^1^. To the best of our knowledge, the association of the volume of “current frequency of shift work” (early in the pandemic) has not been reported; however, the finding is in agreement with job type^31^. Our findings regarding air pollution are also concordant with Travaglio et al.’s findings^32^, though our efforts extended this result to demonstrate their lack of robustness in specific analytic model contexts via VoE.

Our data-driven approach identified primarily sociodemographic-related factors such as “current frequency of shift work” associated with COVID-19 outcomes early in the pandemic. Age and household factors (e.g., as “participant having son and/or daughter in household”, “number of people in participant’s household”) played a more prominent role as the pandemic progressed. Previously reported associations (e.g., air pollution) are sensitive to both time of recruitment of patients at risk and time of COVID-19 test, in addition to modeling assumptions, such as adjustment variable selection.

It is also important to note the limitations of our study. The UKB cohort is not a representative sample of the general UK population as many participants are less deprived socioeconomically, generally healthier overall, and are predominantly White Caucasian^13,29^. Others have shown that associations may also be affected by collider bias^33^. More specifically, our positivity outcome results may be biased by sampling and public health testing strategy. Additionally, there are unbalanced sample sizes corresponding to the two time periods, which might explain some of the variation of the exposure effects between the two time periods. Also, many exposures are unlikely to be available for all participants in the entire cohort. For example, the exposure category of infectious antigens (e.g., “1gG antigen for Herpes Simplex virus-1”) has the highest missingness rate of ~98%. Additionally, we did not consider nonlinear associations. Also, we did not have the opportunity to consider confounders for each exposure or group of exposures as there is no consensus for what variables may be considered as ‘confounders’ for such associations. Lastly, our study considered only 14 disease and health conditions but comorbidity burden taken as a whole has been reported in the literature to play a significant role in risk for COVID-19 positivity (among other COVID-19 related outcomes)^34^. Moreover, cumulative comorbidity burden may mediate some of the age associations^35–37^. Future work should consider the correlated effects of comorbidity burden towards the stabilization (or lack thereof) of the laboratory parameters (blood biomarkers).

Our results suggest that COVID-19 observational associations are dependent on the time of pandemic and public health priorities need to be nimble to changing risk as the pandemic progresses.

## Supplementary information

Description of Additional Supplementary Data Files

Supplementary Data 1

Supplementary Data 2

Supplementary Data 3

Supplementary Data 4

Supplementary Data 5

Supplementary Data 6

Supplementary Data 7

Supplementary Data 8

Supplementary Data 9

Supplementary Data 10

Supplementary Information

Reporting Summary

## Data Availability

The data from the UK Biobank that support the findings of this study are available upon application (https://www.ukbiobank.ac.uk/register-apply/). Data for generating figures is available on Figshare^38–40^. The source data for Figs. 1 and 4 is stored at 10.6084/m9.figshare.21909726.v1^38^ and 10.6084/m9.figshare.21909408.v4^39^. Source data for Figs. 2 and 3 can be found at 10.6084/m9.figshare.21909408.v4^39^ and 10.6084/m9.figshare.21909711.v1^40^, respectively, Summary statistics (including risk ratios, FDR-corrected *p*-values, sample sizes, etc.) can be found in Supplementary Data 1-10. More specifically, Supplementary Data 1 and Supplementary Data 2 contain means, standard deviations, and proportions. Supplementary Data 3 contains risk ratios (and 95% CIs), *p*-values, and FDR-corrected *p*-values. Supplementary Data 4, 5, 7, and 8 contain risk ratios (and 95% CIs), *p*-values, FDR-corrected *p*-values, and sample sizes. Supplementary Data 6 contains delta risk ratios and FDR-corrected *p*-values. Supplementary Data 9 and 10 contain proportions.

## Code availability

We performed all analyses using R version 3.6.1. We made our code accessible at (https://github.com/stejat98/UKB_COVID_XWAS) and on Zenodo (10.5281/zenodo.7542752)^23^.

## References

[1] Chadeau-Hyam, M. et al. Risk factors for positive and negative COVID-19 tests: a cautious and in-depth analysis of UK biobank data. *Int. J. Epidemiol*. 10.1093/ije/dyaa134 (2020). 10.1093/ije/dyaa134 PMC7454561 32814959

[2] Niedzwiedz CL (2020). Ethnic and socioeconomic differences in SARS-CoV-2 infection: prospective cohort study using UK Biobank. BMC Med..

[3] Roso-Llorach, A. et al. Evolving mortality and clinical outcomes of hospitalized subjects during successive COVID-19 waves in Catalonia, Spain. *Glob. Epidemiol.* **4**, 100071 (2022). 10.1016/j.gloepi.2022.100071 PMC8739818 35018339

[4] Patel CJ (2022). The demographic and socioeconomic correlates of behavior and HIV infection status across sub-Saharan Africa. Commun. Med..

[5] Patel CJ, Bhattacharya J, Ioannidis JPA, Bendavid E (2018). Systematic identification of correlates of HIV infection: an X-wide association study. AIDS.

[6] Patel CJ, Ioannidis JPA (2014). Studying the elusive environment in large scale. JAMA.

[7] Ioannidis JPA, Loy EY, Poulton R, Chia KS (2009). Researching genetic versus nongenetic determinants of disease: a comparison and proposed unification. Sci. Transl. Med..

[8] Patel CJ, Bhattacharya J, Butte AJ (2010). An Environment-Wide Association Study (EWAS) on type 2 diabetes mellitus. PLoS One.

[9] McGinnis DP, Brownstein JS, Patel CJ (2016). Environment-Wide Association Study of Blood Pressure in the National Health and Nutrition Examination Survey (1999-2012). Sci. Rep..

[10] Patel CJ (2013). Systematic evaluation of environmental and behavioural factors associated with all-cause mortality in the United States national health and nutrition examination survey. Int. J. Epidemiol..

[11] Patel CJ, Burford B, Ioannidis JPA (2015). Assessment of vibration of effects due to model specification can demonstrate the instability of observational associations. J. Clin. Epidemiol..

[12] Sudlow C (2015). UK biobank: an open access resource for identifying the causes of a wide range of complex diseases of middle and old age. PLoS Med..

[13] Fry, A. et al. Comparison of Sociodemographic and Health-Related Characteristics of UK Biobank Participants With Those of the General Population. *Am. J. Epidemiol.* **186**, 1026–1034 (2017). 10.1093/aje/kwx246 PMC5860371 28641372

[14] Armstrong, J. et al. Dynamic linkage of COVID-19 test results between Public Health England’s Second Generation Surveillance System and UK Biobank. *Microb. Genom.* **6**. Preprint at 10.1099/mgen.0.000397 (2020). 10.1099/mgen.0.000397 PMC7478634 32553051

[15] McCaw, Z. R., Lane, J. M., Saxena, R., Redline, S. & Lin, X. Operating characteristics of the rank-based inverse normal transformation for quantitative trait analysis in genome-wide association studies. *Biometrics*10.1111/biom.13214 (2019). 10.1111/biom.13214 PMC8643141 31883270

[16] Millard LAC, Davies NM, Gaunt TR, Davey Smith G, Tilling K (2018). Software Application Profile: PHESANT: a tool for performing automated phenome scans in UK Biobank. Int. J. Epidemiol..

[17] Zou, G. A Modified Poisson Regression Approach to Prospective Studies with Binary Data. *Am. J. Epidemiol.* **159**, 702–706 (2004). 10.1093/aje/kwh090 15033648

[18] Mansournia MA, Nazemipour M, Naimi AI, Collins GS, Campbell MJ (2021). Reflection on modern methods: demystifying robust standard errors for epidemiologists. Int. J. Epidemiol..

[19] Benjamini, Y. & Hochberg, Y. Controlling the False Discovery Rate: A Practical and Powerful Approach to Multiple Testing. *J. R. Stat. Soc. Series B.* **57**, 289–300 (1995).

[20] Williamson EJ (2020). Factors associated with COVID-19-related death using OpenSAFELY. Nature.

[21] Wu, Y. et al. Genome-wide association study of medication-use and associated disease in the UK Biobank. *Nat. Commun.* **10**, 1891 (2019). 10.1038/s41467-019-09572-5 PMC6478889 31015401

[22] Klau, S., Hoffmann, S., Patel, C. J., Ioannidis, J. P. A. & Boulesteix, A.-L. Examining the robustness of observational associations to model, measurement and sampling uncertainty with the vibration of effects framework. *Int. J. Epidemiol.* **50**, 266–278 (2021). 10.1093/ije/dyaa164 PMC7938511 33147614

[23] stejat. *stejat98/UKB_COVID_XWAS: v1.0.0*. (Zenodo, 2023). 10.5281/ZENODO.7542752.

[24] Daghlas, I. et al. Selection into shift work is influenced by educational attainment and body mass index: a Mendelian randomization study in the UK Biobank. *Int. J. Epidemiol.* **50**, 1229–1240 (2021). 10.1093/ije/dyab031 PMC8562336 33712841

[25] Tierney BT (2021). Leveraging vibration of effects analysis for robust discovery in observational biomedical data science. PLoS Biol..

[26] Rogers, A. E., Hwang, W.-T., Scott, L. D., Aiken, L. H. & Dinges, D. F. The Working Hours Of Hospital Staff Nurses And Patient Safety. *Health Affairs* **23**, 202–212 (2004). 10.1377/hlthaff.23.4.202 15318582

[27] Tapela N (2021). Original research: Prevalence and determinants of hypertension control among almost 100 000 treated adults in the UK. Open Heart.

[28] COVID-19 Host Genetics Initiative. Mapping the human genetic architecture of COVID-19. *Nature*10.1038/s41586-021-03767-x (2021). 10.1038/s41586-021-03767-x PMC8674144 34237774

[29] Hastie CE (2020). Vitamin D concentrations and COVID-19 infection in UK Biobank. Diabetes Metab. Syndr..

[30] Hernández, J. L. et al. Vitamin D Status in Hospitalized Patients with SARS-CoV-2 Infection. *J. Clin. Endocrinol. Metab*. 10.1210/clinem/dgaa733 (2020). 10.1210/clinem/dgaa733 PMC7797757 33159440

[31] Mutambudzi, M. et al. Occupation and risk of severe COVID-19: prospective cohort study of 120 075 UK Biobank participants. *Occup. Environ. Med.* **78**, 307–314 (2020). 10.1136/oemed-2020-106731 PMC7611715 33298533

[32] Travaglio, M. et al. Links between air pollution and COVID-19 in England. *Environ. Pollut.* **268**, 115859 (2021). 10.1016/j.envpol.2020.115859 PMC7571423 33120349

[33] Griffith, G. J. et al. Collider bias undermines our understanding of COVID-19 disease risk and severity. *Nat. Commun.* **11**, 5749 (2020). 10.1038/s41467-020-19478-2 PMC7665028 33184277

[34] Wynants L (2020). Prediction models for diagnosis and prognosis of covid-19: systematic review and critical appraisal. BMJ.

[35] Monterde D (2021). Performance of Three Measures of Comorbidity in Predicting Critical COVID-19: A Retrospective Analysis of 4607 Hospitalized Patients. Risk Manag. Healthc. Policy.

[36] Vela E (2022). Development and validation of a population-based risk stratification model for severe COVID-19 in the general population. Sci. Rep..

[37] Ho FK (2020). Is older age associated with COVID-19 mortality in the absence of other risk factors? General population cohort study of 470,034 participants. PLoS One.

[38] Tangirala, S. COVID-19 Positivity results data for Figs. 1and 4. 10.6084/M9.FIGSHARE.21909726.V1 (2023).

[39] Tangirala, S. COVID-19 Positivity results data for Fig. 2. 10.6084/M9.FIGSHARE.21909408.V4 (2023).

[40] Tangirala, S. COVID-19 Positivity results data for Fig. 3. 10.6084/M9.FIGSHARE.21909711.V1 (2023).

