# Supplementary material for: Prioritization of COVID-19 risk factors in July 2020 and February 2021 in the UK
Source: Commun Med (Lond). 2023 Mar 30;3:45. doi: 10.1038/s43856-023-00271-3 (PMC10062272; doi:10.1038/s43856-023-00271-3)
Supplement: Supplementary file 1 — Description of Additional Supplementary Data Files [file 43856_2023_271_MOESM1_ESM.pdf]

Description of Additional Supplementary Files

File Name: Supplementary Data 1

Description: Baseline characteristics of UK Biobank (UKB) cohort participants.

File Name: Supplementary Data 2

Description: Baseline characteristics of UKB participants sampled in 02/02/2021 and 7/17/2020.

File Name: Supplementary Data 3

Description: Top baseline demographic associations for COVID-19 positivity for second time point (tests between 07/18/2020 and 02/02/21).

File Name: Supplementary Data 4

Description: Top exposures (FDR-corrected p-values in top 10 %) associated with COVID-19 positivity for first time point (tests until 07/17/2020).

File Name: Supplementary Data 5

Description: Top exposures (FDR-corrected p-values in top 10 %) associated with COVID-19 positivity for second time point (tests between 07/18/2020 and 02/02/21).

File Name: Supplementary Data 6

Description: Top exposures with interaction effect (FDR-corrected p-values in top 10 %) with timepoint.

File Name: Supplementary Data 7

Description: Top exposures (FDR-corrected p-values in top 10 %) associated with COVID-19 hospitalization for first time point (tests until 07/17/2020).

File Name: Supplementary Data 8

Description: Top exposures (FDR-corrected p-values in top 10 %) associated with COVID-19 hospitalization for second time point (tests between 07/18/2020 and 02/02/21).

File Name: Supplementary Data 9

Description: Proportion of participants in each family income category for each assessment center for first time point (tests until 07/17/2020).

File Name: Supplementary Data 10

Description: Proportion of participants in each family income category for each assessment center for second time point (tests between 07/18/2020 and 02/02/21).
