# Supplementary material for: Prioritization of COVID-19 risk factors in July 2020 and February 2021 in the UK
Source: Commun Med (Lond). 2023 Mar 30;3:45. doi: 10.1038/s43856-023-00271-3 (PMC10062272; doi:10.1038/s43856-023-00271-3)
Supplement: Supplementary file 12 — Supplementary Information [file 43856_2023_271_MOESM12_ESM.pdf]

Supporting Information

Prioritization of COVID-19 risk factors in July 2020 and February 2021 in the UK

Tangirala et al.

Supplementary Figures

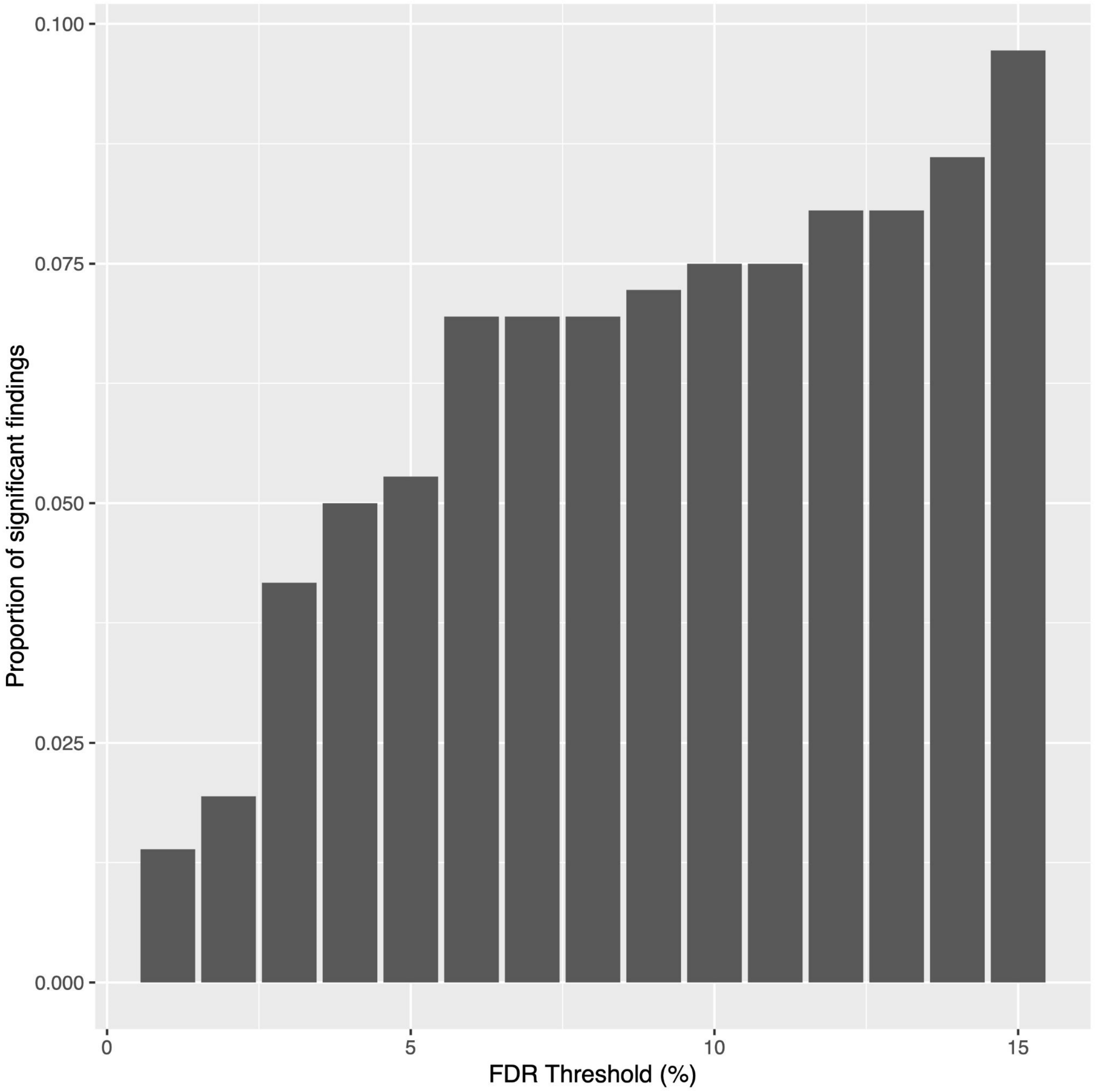

Supplementary Figure 1. Bar plot of proportion of significant findings across a range of FDR-corrected p-value thresholds (tests until 07/17/2020). Bar plot visualizing the proportion of significant findings at FDR-corrected p-value thresholds ranging from 1 to 15% for tested participants until 07/17/2020.

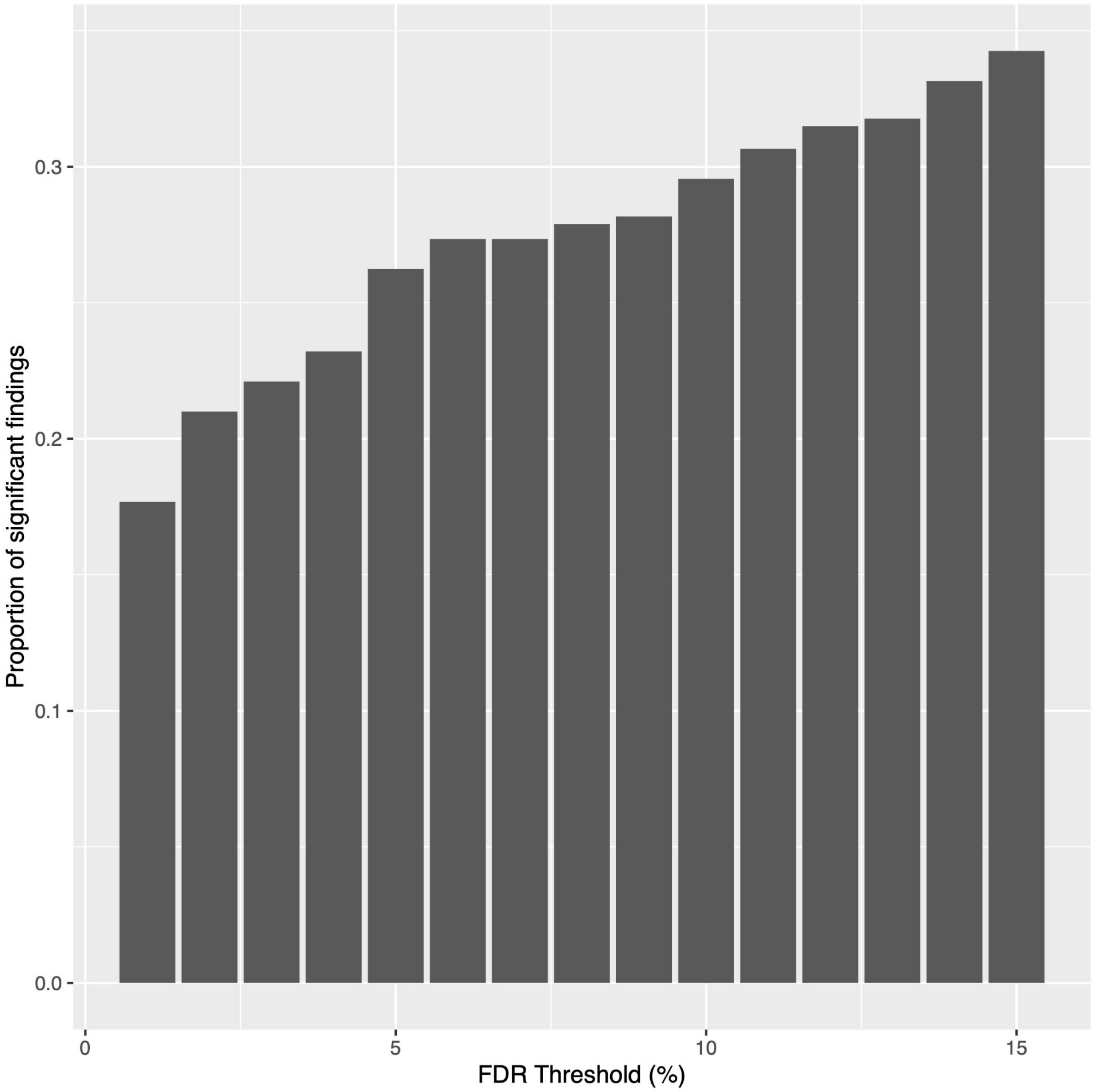

Supplementary Figure 2. Bar plot of proportion of significant findings across a range of FDR-corrected p-value thresholds (tests between 07/18/2020 and 02/02/21). Bar plot visualizing the proportion of significant findings at FDR-corrected p-value thresholds ranging from 1 to 15% for tested participants between 07/18/2020 and 02/02/21.

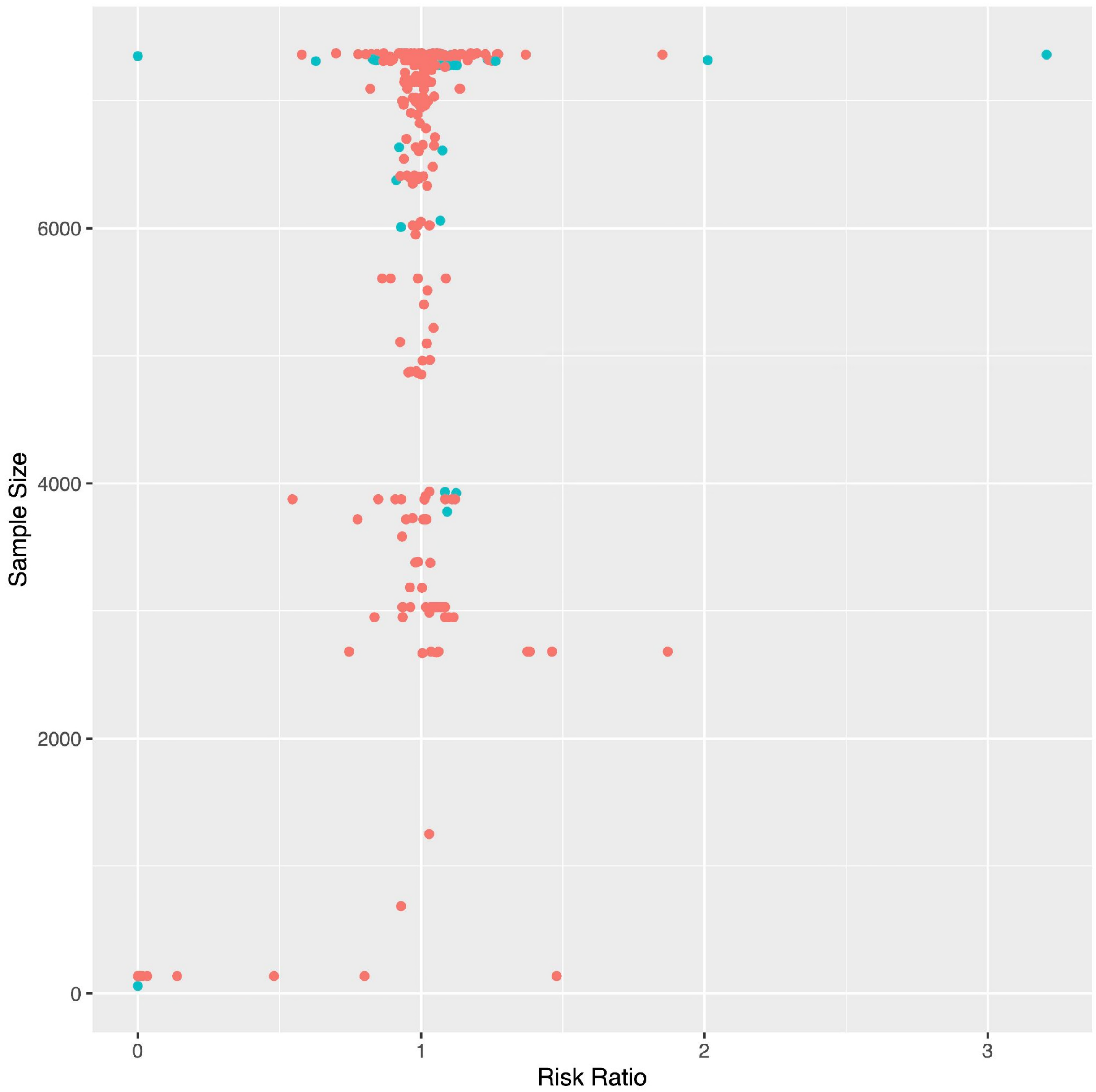

Supplementary Figure 3. Scatterplot of sample size versus risk ratio of COVID-19 positivity associations (tests until 07/17/2020). Scatterplot visualizing the sample size versus risk ratio of COVID-19 positivity associations for tests until 07/17/2020. Associations which have a significant FDR-corrected p-value in the top 10 percentile are colored blue (versus red if not).

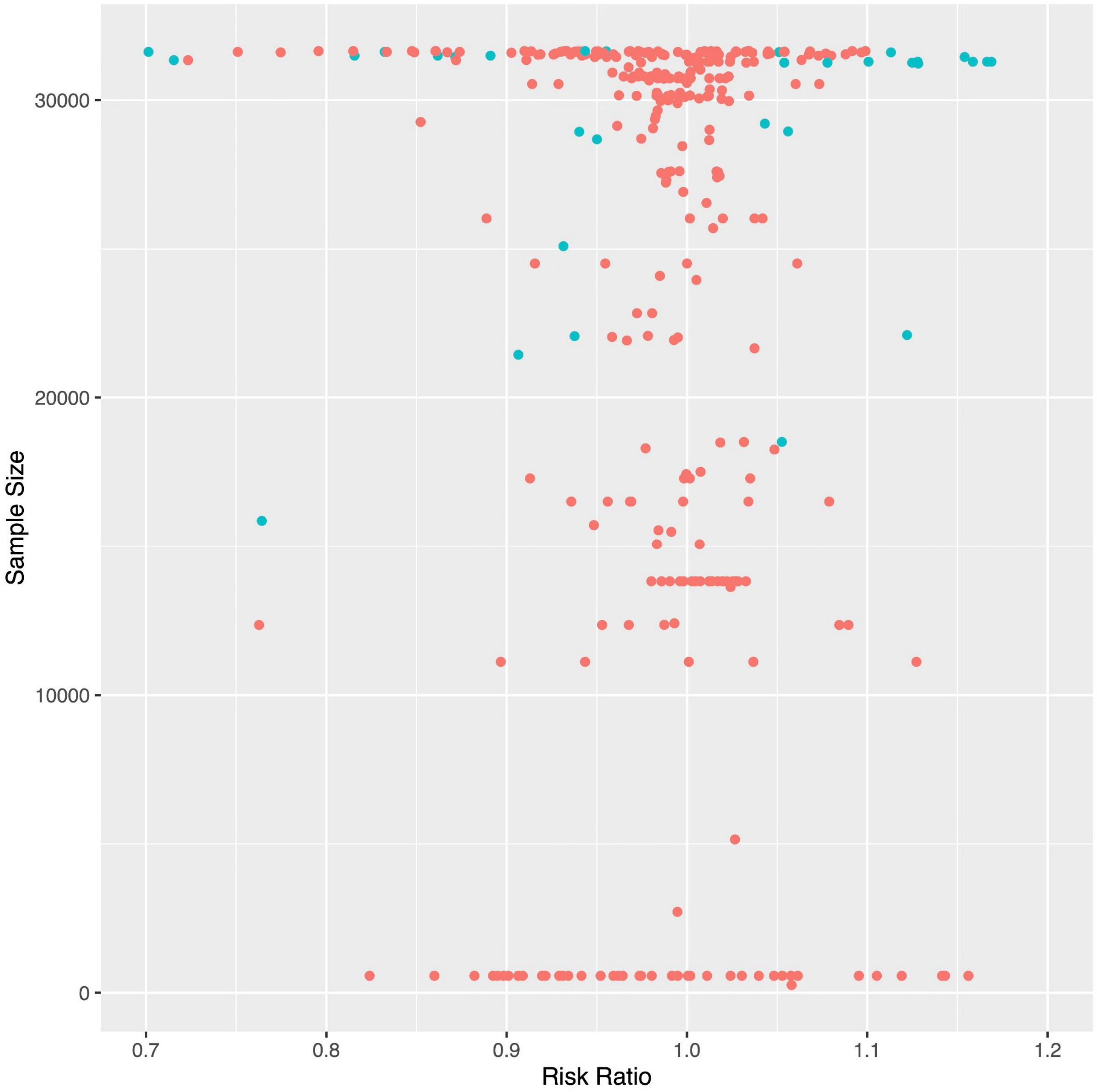

Supplementary Figure 4. Scatterplot of sample size versus risk ratio of COVID-19 positivity associations (tests between 07/18/2020 and 02/02/21). Scatterplot visualizing the sample size versus risk ratio of COVID-19 positivity associations for tests between 07/18/2020 and 02/02/21. Associations which have a significant FDR-corrected p-value in the top 10 percentile are colored blue (versus red if not).

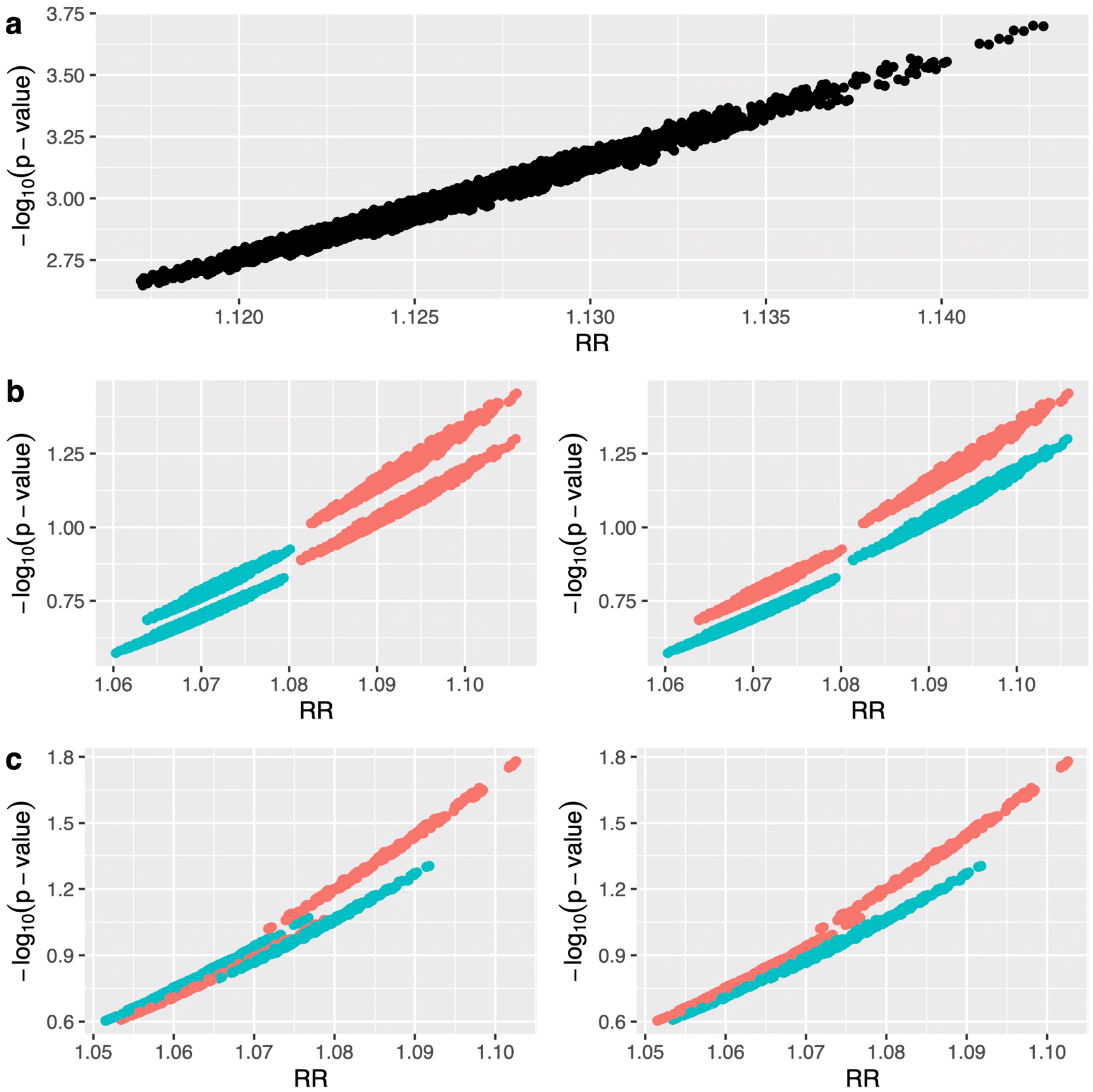

Supplementary Figure 5. Volcano plots of vibration of effects of current frequency of shift work, nitrogen oxide pollution, and BMI with COVID-19 positivity (tests until 07/17/2020). Volcano plots displaying the “vibration” of associations across all adjustment combinations. (a) The risk ratios (RR) versus the negative log (base 10) of p-values for all model combinations including current frequency of shift work. (b) The plot on the left depicts risk ratios (RR) versus the negative log (base 10) of p-values for all model combinations including nitrogen oxide air pollution (colored by inclusion (blue) or exclusion (red) of Townsend deprivation index in the models). The plot on the right depicts risk ratios (RR) versus the negative log (base 10) of p-values for all model combinations including nitrogen oxide air pollution (colored by inclusion (blue) or exclusion (red) of urban (less sparse) home area population density in the models). (c)

The plot on the left depicts risk ratios (RR) versus the negative log (base 10) of p-values for all model combinations including BMI (colored by inclusion (blue) or exclusion (red) of apolipoprotein A in the models). The plot on the right depicts risk ratios (RR) versus the negative log (base 10) of p-values for all model combinations including BMI (colored by inclusion (blue) or exclusion (red) of HDL cholesterol in the models).

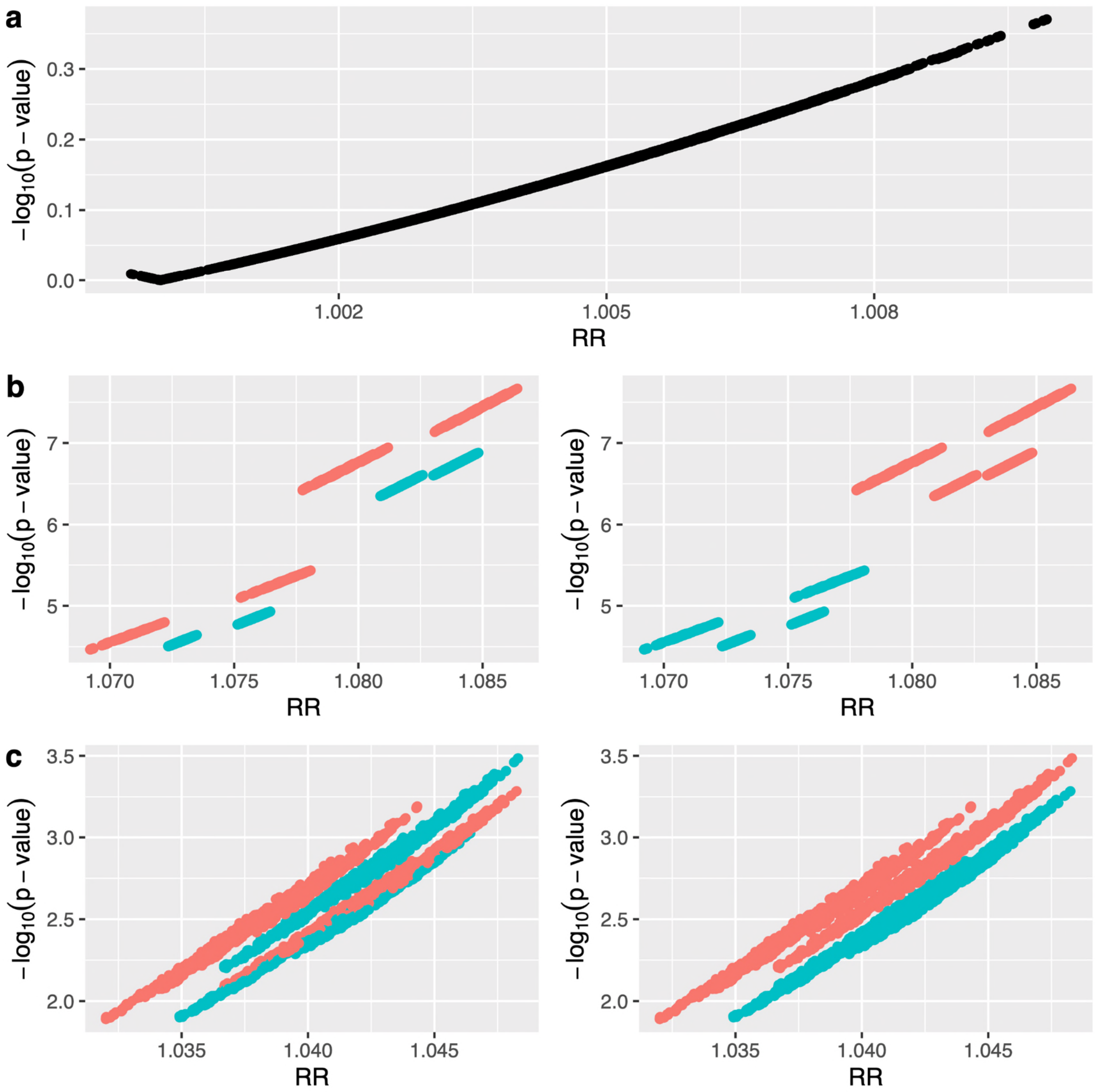

Supplementary Figure 6. Volcano plots of vibration of effects of current frequency of shift work, nitrogen oxide pollution, and BMI with COVID-19 positivity (tests between 07/18/2020 and 02/02/21). Volcano plots displaying the "vibration" of associations across all adjustment combinations. (a) The risk ratios (RR) versus the negative log (base 10) of p-values for all model combinations including current frequency of shift work. (b) The plot on the left depicts risk ratios (RR) versus the negative log (base 10) of p-values for all model combinations including nitrogen oxide air pollution (colored by inclusion (blue) or exclusion (red) of Townsend deprivation index in the models). The plot on the right depicts risk ratios (RR) versus the negative log (base 10) of p-values for all model combinations including nitrogen oxide air pollution (colored by inclusion (blue) or exclusion (red) of urban (less sparse) home area population density in the models). (c) The plot on the left depicts risk ratios (RR) versus the

negative log (base 10) of p-values for all model combinations including BMI (colored by inclusion (blue) or exclusion (red) of apolipoprotein A in the models). The plot on the right depicts risk ratios (RR) versus the negative log (base 10) of p-values for all model combinations including BMI (colored by inclusion (blue) or exclusion (red) of HDL cholesterol in the models).

Supplementary Tables

**Shift work category**	**COVID-19 Prevalence**
Never/rarely	15.20%
Sometimes	22.75%
Usually	21.64%
Always	28.60%

Supplementary Table 1. Distribution of COVID-19 prevalence across shift work categories for first time point (tests until 07/17/2020).

Drug name	UKB Coding	ATC code
methotrexate	1140869848	L04AX03
azathioprine	1140869930	L04AX01
mycophenolate	1140925978	L04AA06
tacrolimus	1140911642	L04AD02
leflunomide	1141166294	L04AA13
Adalimumab	1141188594	L04AB04
Cyclosporine	2038459888	L04AD01
Cyclosporine	1141167744	L04AD01
Cyclosporine	1140909844	L04AD01
Adalimumab	1141188588	L04AB04
Methotrexate	1140910036	L04AX03
Mycophenolate Mofetil	1140925986	L04AA06
Tacrolimus	1141171242	L04AD02
Azathioprine	1141145996	L04AX01
Cyclosporine	1140910382	L04AD01
Azathioprine	1140909864	L04AX01
Mycophenolic Acid	1141200450	L04AA06
Leflunomide	1141166302	L04AA13
Sirolimus	1141173926	L04AA10

Supplementary Table 2. List of immunosuppressant drugs (with name of drug, UKB drug code, and ATC code) from Wu et al..
