# Supplementary material for: Prioritization of COVID-19 risk factors in July 2020 and February 2021 in the UK
Source: Commun Med (Lond). 2023 Mar 30;3:45. doi: 10.1038/s43856-023-00271-3 (PMC10062272; doi:10.1038/s43856-023-00271-3)
Supplement: Supplementary file 13 — Reporting Summary [file 43856_2023_271_MOESM13_ESM.pdf]

nature research

Corresponding author(s): Dr. Chirag J. Patel

Last updated by author(s): 01/17/2023

# Reporting Summary

Nature Research wishes to improve the reproducibility of the work that we publish. This form provides structure for consistency and transparency in reporting. For further information on Nature Research policies, see our Editorial Policies and the Editorial Policy Checklist.

## Statistics

For all statistical analyses, confirm that the following items are present in the figure legend, table legend, main text, or Methods section.

| n/a | Confirmed | |
|---|---|---|
| ☐ | ☒ | The exact sample size ($n$) for each experimental group/condition, given as a discrete number and unit of measurement |
| ☐ | ☒ | A statement on whether measurements were taken from distinct samples or whether the same sample was measured repeatedly |
| ☐ | ☒ | The statistical test(s) used AND whether they are one- or two-sided<br>*Only common tests should be described solely by name; describe more complex techniques in the Methods section.* |
| ☐ | ☒ | A description of all covariates tested |
| ☐ | ☒ | A description of any assumptions or corrections, such as tests of normality and adjustment for multiple comparisons |
| ☐ | ☒ | A full description of the statistical parameters including central tendency (e.g. means) or other basic estimates (e.g. regression coefficient) AND variation (e.g. standard deviation) or associated estimates of uncertainty (e.g. confidence intervals) |
| ☐ | ☒ | For null hypothesis testing, the test statistic (e.g. $F$, $t$, $r$) with confidence intervals, effect sizes, degrees of freedom and $P$ value noted<br>*Give P values as exact values whenever suitable.* |
| ☒ | ☐ | For Bayesian analysis, information on the choice of priors and Markov chain Monte Carlo settings |
| ☒ | ☐ | For hierarchical and complex designs, identification of the appropriate level for tests and full reporting of outcomes |
| ☐ | ☒ | Estimates of effect sizes (e.g. Cohen's $d$, Pearson's $r$), indicating how they were calculated |

*Our web collection on statistics for biologists contains articles on many of the points above.*

## Software and code

Policy information about availability of computer code

| | |
|---|---|
| Data collection | The data from the UK Biobank that support the findings of this study are available upon application (https://www.ukbiobank.ac.uk/register-apply/). |
| Data analysis | We performed all analyses using R version 3.6.1. We made our code accessible at (https://github.com/stejat98/UKB_COVID_XWAS). |

For manuscripts utilizing custom algorithms or software that are central to the research but not yet described in published literature, software must be made available to editors and reviewers. We strongly encourage code deposition in a community repository (e.g. GitHub). See the Nature Research guidelines for submitting code & software for further information.

## Data

Policy information about availability of data

All manuscripts must include a data availability statement. This statement should provide the following information, where applicable:

- Accession codes, unique identifiers, or web links for publicly available datasets
- A list of figures that have associated raw data
- A description of any restrictions on data availability

We performed all analyses using R version 3.6.1. We made our code accessible at (https://github.com/stejat98/UKB_COVID_XWAS). The data from the UK Biobank that support the findings of this study are available upon application (https://www.ukbiobank.ac.uk/register-apply/).

# Field-specific reporting

Please select the one below that is the best fit for your research. If you are not sure, read the appropriate sections before making your selection.

☒ Life sciences ☐ Behavioural & social sciences ☐ Ecological, evolutionary & environmental sciences

For a reference copy of the document with all sections, see nature.com/documents/nr-reporting-summary-flat.pdf

# Life sciences study design

All studies must disclose on these points even when the disclosure is negative.

| | |
|---|---|
| Sample size | We analyzed two non-overlapping subsets of the UK Biobank [UKB] cohort (total n=502,655 participants) for which we had data pertaining to COVID-19 testing for tests administered until July 17, 2020 and tests administered between July 18, 2020 and February 2, 2021. We excluded participants whose ethnicity was not known, yielding samples of 9,268 and 38,837 participants, respectively. |
| Data exclusions | We excluded participants whose ethnicity was not known. |
| Replication | We performed a massive sensitivity analysis (vibration of effects [VoE]) to systematically test how associations vary due to combinations of potential confounders. We were not able to use other cohorts to replicate as (to our knowledge) there is no comparable cohort (in size) that had COVID-19 outcome and environmental data. |
| Randomization | This is not relevant to our study. We performed analyses on observational data from a prospective UK cohort. |
| Blinding | This is not relevant to our study. We performed analyses on observational data from a prospective UK cohort. |

# Reporting for specific materials, systems and methods

We require information from authors about some types of materials, experimental systems and methods used in many studies. Here, indicate whether each material, system or method listed is relevant to your study. If you are not sure if a list item applies to your research, read the appropriate section before selecting a response.

## Materials & experimental systems

| n/a | Involved in the study | |
|---|---|---|
| ☒ | ☐ | Antibodies |
| ☒ | ☐ | Eukaryotic cell lines |
| ☒ | ☐ | Palaeontology and archaeology |
| ☒ | ☐ | Animals and other organisms |
| ☐ | ☒ | Human research participants |
| ☒ | ☐ | Clinical data |
| ☒ | ☐ | Dual use research of concern |

## Methods

| n/a | Involved in the study | |
|---|---|---|
| ☒ | ☐ | ChIP-seq |
| ☒ | ☐ | Flow cytometry |
| ☒ | ☐ | MRI-based neuroimaging |

## Human research participants

Policy information about studies involving human research participants

| | |
|---|---|
| Population characteristics | The UK Biobank is a very large and detailed prospective cohort with over 500,000 participants aged 40–69 years (with a roughly even number of men and women) . |
| Recruitment | Participants living "within a 25-mile radius of any of the 22 assessment centres across the UK were identified from NHS patient registers" (Source: UK Biobank, IJE) . In total, around 9 million individuals were invited to participate of which 500,000 participants were recruited.  (Source: UK Biobank, IJE). Of these individuals, our analyses focuses on the subset for which COVID-19 test data was available (for around 40,000 individuals from the start of the pandemic till February 2, 2021). |
| Ethics oversight | UK Biobank and Harvard University IRB (IRB16-2145) |

Note that full information on the approval of the study protocol must also be provided in the manuscript.
